# A Cross-Sectional Study on the Knowledge of Sexually Transmitted Diseases among Young Adults Living in Albaha, Saudi Arabia

**DOI:** 10.3390/ijerph17061872

**Published:** 2020-03-13

**Authors:** Mohammad A. Albanghali, Basim A. Othman

**Affiliations:** Department of Public Health, Faculty of Applied Medical Sciences, Albaha University, 65779 Albaha, Saudi Arabia; bothman@bu.edu.sa

**Keywords:** sexually transmitted diseases, sexually transmitted disease knowledge questionnaire, STDs, STDs-KQ, young adult

## Abstract

Background: Sexually transmitted diseases (STDs) remain one of the most important health challenges in not only developing countries but also developed countries. Discussing STDs in the Saudi society is considered taboo, as social factors and ethics give rise to many obstacles. This study evaluates the knowledge of STDs among young adult students enrolled in Albaha University. Methods: This research is a cross-sectional study involving 1902 young adult students registered at Albaha University. STDs knowledge scores (STDs-KSs) were calculated using a predesigned and validated STDs knowledge questionnaire with 27 items adapted from previously developed questionnaires. Results: The estimated overall mean of STDs-KS was 7.95 ± 4.29. Female participants showed a significantly higher mean of STDs-KS, compared to males (8.51 ± 4.14 vs. 7.32 ± 4.38, *p* < 0.0001). Participants registered in health sciences programs showed higher STDs-KS, compared to participants from arts and sciences programs (*p* < 0.0001). Conclusions: Evidence from this study suggests a lack of STDs knowledge among young adults. To promote STDs awareness among this population, more health educational programs should be included in school curricula at the late stages of secondary education.

## 1. Introduction

Sexually transmitted diseases (STDs) remain one of the most important health challenges in not only developing countries, but also developed countries. In Saudi Arabia, research reports that in period between 2005 and 2012 there were 68,886 new cases of STDs. Of these cases, acquired immunodeficiency syndrome (AIDS) patients consist of around 14.3% (n = 9843, with an approximate average of 1406 new cases per year) of all cases, whereas non-gonococcal urethritis cases are the highest STDs, which consist of 51.7%. This is followed by trichomoniasis (18.4%), gonococcal urethritis (4.4%), syphilis (2.6%), genital warts (5.8%), genital herpes (2.2%), and chancroid (0.7%). Furthermore, there is inadequate research and reports in current literature to estimate incidence rates of STDs among Saudis over the past few years [1]. The main problem with these types of diseases is that patients can be asymptomatic and remain undiagnosed. Information and dissemination campaigns are considered one of the hallmarks of preventive medicine. In 2009, the World Health Organization highlighted the importance of a comprehensive STDs control strategy, which includes the promotion and provision of prevention strategies, targeted community-based interventions, reliable data to guide the response, as well as effective clinical services for STD patients. Moreover, the World Health Organization emphasizes the value of knowledge and awareness of the population, as this promotes a drop in the rate of STD incidences and prevalence in the long-run [2]. Research indicates overall low levels of awareness and knowledge of STDs among people in Europe, Africa, and the Middle East; however, evidence from previous research suggests that the majority of people showed the highest awareness and knowledge of AIDS compared to another STDs [3,4,5,6,7,8,9].

Regardless of age, STDs remain a sensitive topic, and discussing STDs in the Saudi society is considered taboo, where social factors and ethics give rise to many obstacles, such as; social desirability bias and fear of judgement. People who live in a conservative society are more likely to be denied STD knowledge. Thus, limited information about awareness and attitude towards STDs were available from these populations [7,10].

The last official population census, in 2010, reported 348,636 Saudi citizens live in seven provinces, namely: Albaha, Baljurashi, Almandaq, AlMakhwah, AlAqeeq, Qilwah, and Alqura of the Albaha region, and 74,848 citizens from this population are young adults (45% male and 55% female) aged between 20 and 30 years [11]. Among these young adults, more than 26,000 persons were registered at Albaha University as undergraduate students, which is about 35% of the total population of young adults living in Albaha. This study aims to evaluate the knowledge of STDs among young adults enrolled in Albaha University.

## 2. Materials and Methods

### 2.1. Participants and Settings

The setting for this study was the Albaha University, Saudi Arabia, which had approximately 26,000 young adults of the Albaha population registered as undergraduates in three different streams (including health sciences, arts, and sciences). These streams were distributed among five campuses in different provinces within the Albaha region, namely: Albaha, Baljurashi, Almandaq, AlMakhwah, and Qilwah campuses. Of the total number of university students, 1902 students enrolled in 14 different faculties were involved in this simple random cross-sectional study. The recruitment of participants and data collection were carried out between February and May 2019. 

University students were approached by members of the research team and given a brief introduction about the study. All subjects who voluntarily agreed to participate in this study were given a predesigned electronic-based self-explanatory questionnaire. Each participant was given enough time to go through the questionnaire and answer all questions as appropriate. 

### 2.2. Sexually Transmitted Disease Knowledge Questionnaire

The questionnaire designed for the study consisted of two key sections. The first section focused on sociodemographic information, including gender, age, marital status, and the program and faculty the student was enrolled in. The second section of the questionnaire consisted of a set of questions adapted from a previously developed and published questionnaire—the sexually transmitted disease knowledge questionnaire (STDs-KQ), which was first developed and applied by Jaworski and Carey in 2007 [12]. The STDs-KQ had 27 items. The questionnaire was translated from English to Arabic and was used to evaluate the knowledge of STDs among the targeted population of this study. The translation of the STDs-KQ questionnaire was carried out in three phases: First, the two authors of this study independently translated the English version. Second, a panel meeting was conducted to assess and merge the two versions of the translated questionnaire. Finally, the merged version of the questionnaire was reviewed and validated by assigned native Arabic-speaking translators from the English Center at the University (see Appendix A, Table A1). Furthermore, as the translated version of the questionnaire was to be used for the first time in research, and in order to examine the psychometric properties of the new Arabic version of the questionnaire, the entire sample of the 1902 study participants was used to examine the reliability of the questionnaire. The estimated standardized Cronbach’s alpha was 0.74, which demonstrated an acceptable internal consistency in terms of reliability among the 27 items of the Arabic version of the STDs-KQ (see Appendix A, Table A2 and Table A3). For each participant, an STDs knowledge score (STDs-KS) was calculated by adding the number of correctly answered questions out of the 27 items included in the second section of the questionnaire. Thus, the STDs-KS ranged from 0 to 27. For comparison purposes, the calculated mean of the STDs-KS was utilized to classify participants into two groups—namely, low and high STDs-KS.

### 2.3. Statistical Analysis

Data processing and analysis were carried out using the Statistical Package for the Social Sciences ® software (version 20.0) (IBM, Armonk, NY). Independent sample t-test, one-way ANOVA test, Chi-square test and Fisher’s exact test were used for the comparison, as appropriate. Logistics regression analysis was used to explore the association between STDs-KS and different observed factors. In terms of reliability and validity, standardised Cronbach’s alpha was estimated and reported for examining the psychometric properties of the translated version of STDs-KQ. A *p*-value of 0.05 was considered a significance level for all the statistical tests.

### 2.4. Ethical Approval

Ethical approval for this study was obtained from the deanship of scientific research at Albaha University with reference number (1440-31-40190688). 

## 3. Results

### 3.1. Participants’ Characteristics

One thousand nine hundred two participants were involved in this study. The proportions of male (47%) and female (53%) involved in this study did not differ significantly. The majority of the participants (92%) were 18–23 years old, only a few (8%) were above 23 years. Almost all the participants were single (93%) and only 6.5% were married. About half of the participants (57%) were registered in arts programs, 15% were registered in health sciences programs, and 28% in sciences programs (see Table 1).

### 3.2. Knowledge of Sexually Transmitted Disease

**Figure 1:**
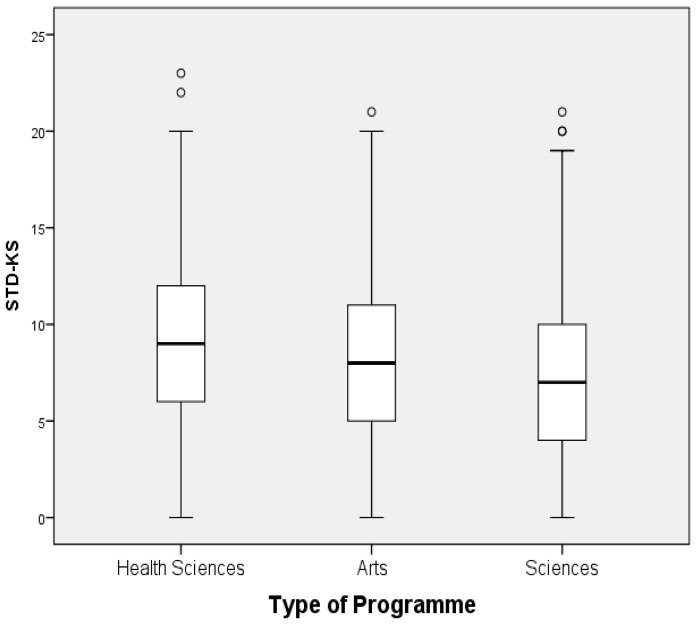
Box-plot of sexually transmitted disease knowledge scores by types of program.

**Figure 2:**
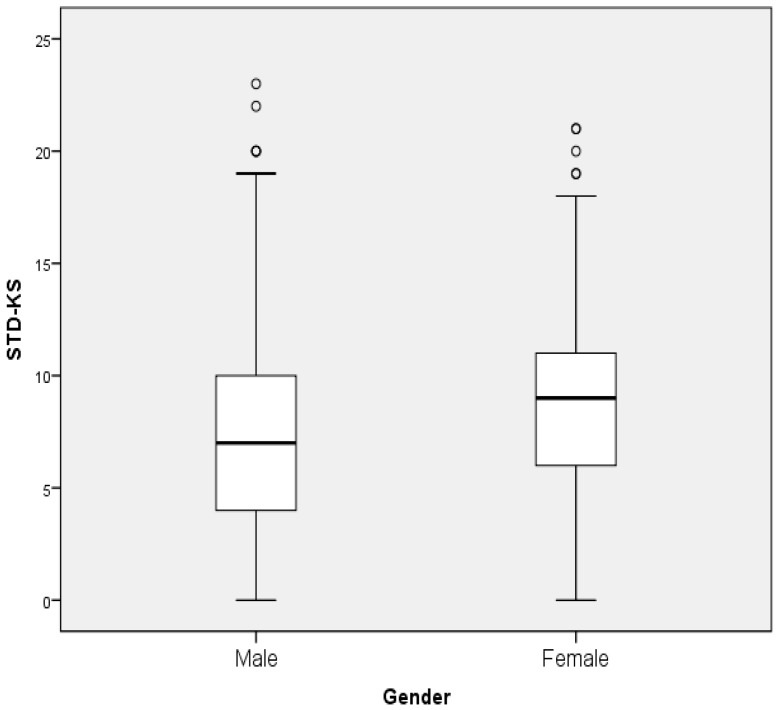
Box-plot of sexually transmitted disease knowledge scores by gender.

The overall mean and standard deviation of STDs-KS was 7.95 ± 4.29. Female participants showed a significantly higher mean of STDs-KS compared to males (8.51 ± 4.14 vs. 7.32 ± 4.38, *p* < 0.0001) (see Table 2, Figure 1 and Figure 2). Median (interquartile range) for STDs-KS was 8 (5–11). Analysis indicates that the type of program in which the participant was registered, and gender, have a significant influence on the STDs-KS (*p* < 0.0001). Logistic regression analysis was used to confirm evidence of the association between gender, type of program, and STDs-KS with estimated *p* < 0.0001; (female: odds ratio (OR) = 1.68, 95% CI = 1.38–2.04), (health sciences program: OR = 1.88, 95% CI = 1.4–2.52, and arts program: OR = 1.14, 95% CI = 0.92–1.42); hence, no adjustment was made to other factors (age, marital status and provinces).

## 4. Discussion

Knowledge of STDs is considered the first line of defense to minimize the STD incidence rate and maintain the wellbeing of the population. Therefore, this study was designed to investigate the knowledge of STDs among young adults living in the Albaha region in Saudi Arabia and registered as undergraduate students in Albaha University. Findings reported in this study were consistent with evidence from previous literature [4,13,14,15]. Thus, the current work supports the suggestion that further effort and sexual education program are required to increase and advance knowledge of STDs among young adults of many population.

Female participants showed higher STDs-KS compared to male students, which is consistent with few previous studies [4,13]. The question remains: is the risk of accruing and transmitting STDs among Saudis male and female is equal? Due to lack of published data from authorities, research on incidence rate, and data on prevalence of STDs among Saudis, answering such a question remains problematic. Regardless of gender, several studies have highlighted the lack of knowledge and awareness among different populations living in Saudi [4,14,15,16], whereas, evidence suggest a low prevalence rate of STDs among Saudis [1,17,18]. Two factors may positively impact the prevalence of STDs among Saudis—the conservative cultural nature of the society and religion rules, which prohibit intimate contact outside marriage. In addition, evidence suggest that male circumcision, which is an obligatory practice by Islam, have been strongly associated with lower HIV and another STDs prevalence [19,20]. Furthermore, owing to culture and religion, which may influence behavior of Saudis, future research must consider investigating the association between level of knowledge, practice towards STDs, and risk for acquiring STDs. In addition, fear of judgement and legal percussions are two reasons that may influence estimating the actual prevalence of STDs among citizens and residents of Saudi, as these reasons may result in patients reluctant to seek medical help. Unlike health sciences students, which expected to show the highest STDs-KS due to the nature and content of program curriculum, students registered in art programs showed higher STDs-KS compared to their counterparts in the science programs. This finding required further investigation to determine the actual reason behind such improvement in knowledge level observed from art program students. 

Currently there is no evidence-based health educational program, with a focus on men and women’s health, which applies to and targets the young or adolescents in Saudi. Whereas, evidence in literature suggests a high positive impact associated with applying educational programs for promoting healthy practice for minimizing STD transition [21,22,23]. Sex education is compulsory in several countries; however, there are still countries, such as Saudi, that have not yet applied such educational material at any education level. There was some effort and campaigns within the last few years to educate young students, to protect themselves against sexual and behavioral abuse; however, the need to implement educational programs within curriculum remains at high importance. Educating students at university can be carried by utilizing university compulsory courses. Most Saudi universities apply a set of compulsory courses within all curriculum in different programs, and no student can graduate unless they study these courses; thus, it might be useful to add a well-designed course that focuses on men and women’s health to the list of compulsory courses. Consequently, university students will have a proper chance to build their knowledge of healthy relationships.

Several studies in the literature developed different types of questionnaires aimed at assessing the knowledge and awareness of STDs; however, evidence from such studies may be limited due to I) lack of reliability of developed questionnaires, and II) inability to compare and/or merge outcomes from these studies. Several studies have applied predesigned questionnaires, such as the STDs knowledge questionnaire (STDs-KQ), which was first developed by Jaworski and Carey in 2007 for the purpose of evaluating the knowledge of STDs [12,24,25,26,27]. Applying a standardized or unified questionnaire has several advantages. One of these advantages is it enables combining results and/or comparing outcomes from different studies to estimate awareness and knowledge on a wider population. In addition, such a questionnaire helps to repeatedly measure the awareness of a targeted population with less chances of bias, and allow measuring awareness before and after exposing the targeted population to specific educational interventions [28]. This study managed to create and validate the Arabic version of STDs-KQ, which was developed and published by Jaworski and Carey in 2007 [12]. The new Arabic version assisted in measuring knowledge and awareness in unified methods; thus, provided researchers with a tool that could help merge and combine results from different studies with less chance of bias [12].

The study has a few limitations. Researchers focusing on knowledge of STDs usually combine investigation of knowledge with attitude toward STDs. However, owing to social and cultural reasons, it was difficult to involve attitude and practice towards STDs in this study. Barriers such as social desirability bias and fear of judgement among the local population may, perhaps, negatively influence targeted subjects to take part in this study. Therefore, it was decided that any questions investigating the attitude aspects that may result in harassment for the participants should be excluded. Furthermore, participants involved in this study were only university students. Thus, the generalizability of findings from this study among young adults of the Albaha population might be limited. However, to maximize the representativity of the collected sample, the researchers recruited students (as many as possible) from different provinces of the Albaha region, enrolled in different program types. Future studies must cover a larger sample and involve young adults not registered at university.

## 5. Conclusions

Although female participants show higher STDs-KS than males, the overall knowledge of STDs remains significantly low. Health educational programs focusing on men’s and women’s health, and targeting university students can aid the promotion of STD awareness among this population, especially students registered in arts and science programs.

## Figures and Tables

**Table 1:** Sociodemographic characteristics of the participants.

**Title**	**All Participantsn** **n = 1902**		**Malen** **n = 896 (47%)**		**Femalen** **n = 1006 (53%)**		*p*-value *
**Age (years)**							
18–23	1751	(92.1%)	763	(85.2%)	988	(98.2%)	<0.0001
24–27	151	(7.9%)	133	(14.8%)	18	(1.8%)	
**Marital status**							
Single	1773	(93.2%)	876	(97.77%)	897	(89.2%)	<0.0001
Married	123	(6.5%)	19	(2.12%)	104	(10.3%)	
Divorced	4	(0.2%)	0	(0.00%)	4	(0.4%)	
Widow	2	(0.1%)	1	(0.11%)	1	(0.1%)	
**Type of program**							
Health sciences	280	(14.7%)	173	(19.31%)	107	(10.6%)	<0.0001
Arts	1086	(57.1%)	414	(46.21%)	672	(66.8%)	
Sciences	536	(28.2%)	309	(34.49%)	227	(22.6%)	
**Provinces**							
AlAqeeq	177	(9.3%)	103	(11.50%)	74	(7.4%)	<0.0001
Albaha	863	(45.4%)	380	(42.41%)	483	(48.0%)	
Almandaq	237	(12.5%)	142	(15.85%)	95	(9.4%)	
AlMakhwah	367	(19.3%)	173	(19.31%)	194	(19.3%)	
Alqura	57	(3.0%)	23	(2.57%)	34	(3.4%)	
Baljurashi	154	(8.1%)	39	(4.35%)	115	(11.4%)	
Qilwah	47	(2.5%)	36	(4.02%)	11	(1.1%)	

* Chi-square associated *p*-value calculated for comparison between male and female subjects.

**Table 2:** Association between sexually transmitted disease knowledge score and demographic characteristics of the participants.

**Title**	**Mean ± SD ^†^**	***p*-value**	**STDs-KS ^†^**		**Chi-square**	***p*-value**
			**Low**	**High**		
**Age (years)**						
18–23	7.97 ± 4.29	0.508	966 (55%)	785 (44%)	0.456	0.551
24–27	7.73 ± 4.35		79 (52%)	72 (48%)		
**Gender**						
Male	7.32 ± 4.38	< 0.0001	544 (61%)	352 (39%)	22.8	<0.0001
Female	8.51 ± 4.14		501 (49.8%)	505 (50.2%)		
**Marital status**						
Single	7.87 ± 4.25	0.065	987 (56%)	786 (44%)	5.63	0.073 *
Married	9.21 ± 4.73		55 (45%)	68 (55%)		
Divorced	5.25 ± 4.99		2 (50%)	2 (50%)		
Widow	10.0 ± 4.24		1 (50%)	1 (50%)		
**Type of program**						
Health sciences	9.10 ± 4.49	< 0.0001	127 (45%)	153 (55%)	16.981	< 0.0001
Arts	8.03 ± 4.06		594 (55%)	492 (45%)		
Sciences	7.19 ± 4.49		324 (60%)	212 (40%)		
**Provinces**						
AlAqeeq	7.62 ± 4.34	0.617	97 (55%)	80 (45%)	8.691	0.192
Albaha	8.08 ± 4.46		454 (53%)	409 (47%)		
Almandaq	8.22 ± 3.74		127 (54%)	110 (46%)		
AlMakhwah	7.83 ± 4.25		206 (56%)	161 (44%)		
Alqura	7.42 ± 4.34		38 (76%)	19 (24%)		
Baljurashi	7.79 ± 4.32		95 (62%)	59 (38%)		
Qilwah	7.57 ± 3.95		28 (60%)	19 (40%)		

^†^ SD stands for standard deviation, STDs-KS refers to sexually transmitted disease knowledge score; the cut-off mark utilized to stratify STDs- KS was eight scores, * Fisher’s exact test was used for this comparison.

## Appendix A

**Table A1:** Arabic-translated version of the sexually transmitted disease knowledge questionnaire developed and applied by Jaworski and Carey in 2007 [12].

No.	Items
1	الفيروس الذي يسبب هربس الأعضاء التناسلية هو فيروس الإيدز؟
2	التهابات الجهاز البولي المتكررة يمكن أن تسبب الكلاميديا؟
3	هناك علاج لمرض السيلان؟
4	من السهل الإصابة بفيروس الإيدز إذا كان الشخص مصاباً بأحد الأمراض الأخرى التي تنتقل عن طريق الاتصال الجنسي؟
5	الفيروس الذي يسبب الورم الحليمي البشري هو نفس فيروس الإيدز؟
6	ممارسة الجنس الشرجي تزيد من خطر الإصابة بالتهاب الكبد النوع ب؟
7	بعد فترة قصيرة من الإصابة بفيروس الإيدز، تتطور تقرحات مفتوحة على الأعضاء التناسلية للمصاب؟
8	هناك علاج للكلاميديا؟
9	المرأة المصابة بهربس الأعضاء التناسلية يمكن أن تنقل العدوى إلى طفلها أثناء الولادة؟
10	يمكن للمرأة أن تتعرف على إصابتها بمرض السيلان بالنظر إلى جسدها؟
11	فيروس واحد هو المسبب لجميع الأمراض المنقولة جنسيا؟
12	فيروس الورم الحليمي البشري يستطيع أن يسبب البثور التناسلية؟
13	استخدام الواقي الطبيعي (المصنوع من مواد حيوية مثل أمعاء الحيوان) يمكن أن يحمي الشخص من الإصابة بفيروس نقص المناعة البشرية؟
14	يمكن أن يؤدي فيروس الورم الحليمي البشري إلى الإصابة بالسرطان عند النساء؟
15	الاتصال الجنسي المهبلي هو الوسيلة الوحيدة للإصابة بالبثور التناسلية؟
16	الأمراض المنقولة جنسيا يمكن أن تؤدي إلى مشاكل صحية عادة ما تكون أكثر تأثيراً على الرجال مقارنةً بالنساء؟
17	يمكن للمرأة أن تحدد إصابتها الكلاميديا إذا وجدت رائحة كريهة من الأعضاء التناسلية؟
18	إذا كانت نتيجة اختبار فيروس الإيدز إيجابية، يمكن لهذا الاختبار أن يحدد شدة ظهور أعراض المرض؟
19	هناك لقاح للوقاية من الإصابة بمرض السيلان؟
20	يمكن للمرأة أن تشعر حين إصابتها بالأمراض الجنسية؟
21	يشترط لانتقال الهربس التناسلي لشريك الممارسة الجنسية وجود تقرحات مفتوحة؟
22	هناك لقاح للوقاية من الإصابة بالكلاميديا؟
23	يمكن للرجل أن يشعر بإصابته بالتهاب الكبد النوع ب؟
24	إذا أصيب الشخص بمرض السيلان فإنه يكتسب مناعة من تكرار الإصابة؟
25	يمكن أن يسبب فيروس الورم الحليمي البشري مرض الإيدز؟
26	يكتفى بغسل الأعضاء التناسلية بعد الممارسة الجنسية للوقاية من الإصابة بالبثور التناسلية؟
27	هناك لقاح للوقاية من الإصابة بالتهاب الكبد الوبائي ب؟

**Table A2:** Correlation between each item of the sexually transmitted disease knowledge questionnaire.

	Q1	Q2	Q3	Q4	Q5	Q6	Q7	Q8	Q9	Q10	Q11	Q12	Q13	Q14	Q15	Q16	Q17	Q18	Q19	Q20	Q21	Q22	Q23	Q24	Q25	Q26	Q27
Q1	1.00																										
Q2	0.09	1.00																									
Q3	0.04	−0.06	1.00																								
Q4	−0.05	−0.11	0.18	1.00																							
Q5	0.24	0.10	0.03	0.02	1.00																						
Q6	0.01	−0.03	0.10	0.17	0.04	1.00																					
Q7	0.08	0.17	−0.01	−0.09	0.06	0.02	1.00																				
Q8	0.11	0.04	0.22	0.11	0.05	0.15	0.09	1.00																			
Q9	0.05	−0.01	0.14	0.17	0.03	0.22	0.05	0.19	1.00																		
Q10	0.06	0.12	0.01	−0.01	0.17	0.04	0.09	0.10	0.06	1.00																	
Q11	0.29	0.07	0.04	0.06	0.23	0.06	0.10	0.06	0.11	0.12	1.00																
Q12	0.05	0.06	0.10	0.13	0.10	0.21	0.05	0.20	0.17	0.07	0.08	1.00															
Q13	0.08	0.10	0.05	−0.01	0.12	0.05	0.10	0.02	0.02	0.06	0.10	0.05	1.00														
Q14	0.06	0.05	0.09	0.17	0.12	0.19	0.01	0.22	0.23	0.06	0.07	0.29	0.01	1.00													
Q15	0.15	0.15	0.05	0.05	0.23	0.08	0.13	0.09	0.09	0.15	0.23	0.09	0.16	0.12	1.00												
Q16	0.15	0.12	0.01	0.00	0.14	−0.01	0.14	0.04	0.08	0.13	0.16	0.07	0.13	0.06	0.19	1.00											
Q17	0.07	0.19	−0.03	−0.04	0.15	0.01	0.12	0.02	0.03	0.18	0.09	0.06	0.13	0.07	0.19	0.17	1.00										
Q18	0.14	0.15	−0.05	−0.07	0.09	−0.02	0.11	0.03	0.01	0.06	0.11	0.03	0.09	0.01	0.07	0.15	0.17	1.00									
Q19	0.14	0.15	−0.04	−0.04	0.16	0.05	0.19	−0.02	0.06	0.15	0.15	0.03	0.17	0.03	0.15	0.10	0.20	0.17	1.00								
Q20	0.04	0.19	0.00	−0.04	0.07	−0.01	0.13	0.01	0.02	0.13	0.02	0.01	0.12	−0.01	0.14	0.09	0.15	0.19	0.16	1.00							
Q21	0.12	0.17	−0.01	0.00	0.13	0.07	0.16	0.07	0.04	0.13	0.11	0.10	0.11	0.05	0.18	0.11	0.23	0.11	0.19	0.20	1.00						
Q22	0.06	0.16	−0.01	−0.06	0.09	0.00	0.14	0.03	0.04	0.14	0.08	0.06	0.12	0.05	0.11	0.09	0.12	0.12	0.24	0.15	0.17	1.00					
Q23	0.11	0.17	0.02	−0.01	0.12	0.02	0.11	0.08	0.01	0.14	0.11	0.04	0.10	0.05	0.18	0.10	0.17	0.14	0.13	0.17	0.12	0.21	1.00				
Q24	0.15	0.14	−0.02	0.06	0.15	0.05	0.10	0.03	0.06	0.17	0.16	0.06	0.10	0.07	0.21	0.14	0.18	0.12	0.17	0.14	0.17	0.19	0.18	1.00			
Q25	0.17	0.14	0.01	−0.04	0.22	0.01	0.11	0.04	0.03	0.13	0.19	0.06	0.13	0.10	0.15	0.14	0.12	0.13	0.20	0.12	0.09	0.13	0.15	0.15	1.00		
Q26	0.17	0.06	0.10	0.05	0.16	0.07	0.09	0.04	0.09	0.14	0.24	0.08	0.12	0.04	0.18	0.16	0.10	0.09	0.14	0.13	0.11	0.10	0.12	0.17	0.15	1.00	
Q27	0.10	0.05	0.19	0.15	0.08	0.15	0.01	0.25	0.21	0.06	0.13	0.21	0.02	0.21	0.11	0.07	0.00	0.01	−0.01	0.06	0.01	0.02	0.04	0.07	0.05	0.13	1.00

**Table A3:** Reliability analysis: Corrected item-total correlation of the sexually transmitted disease knowledge questionnaire and Cronbach’s alpha coefficients.

Questions	Scale Mean if Item Deleted	Cronbach’s Alpha if Item Deleted
Q1	7.67	0.730
Q2	7.75	0.733
Q3	7.44	0.741
Q4	7.36	0.744
Q5	7.65	0.728
Q6	7.54	0.737
Q7	7.78	0.734
Q8	7.61	0.733
Q9	7.56	0.733
Q10	7.72	0.731
Q11	7.57	0.727
Q12	7.63	0.731
Q13	7.71	0.734
Q14	7.58	0.732
Q15	7.66	0.724
Q16	7.71	0.731
Q17	7.74	0.730
Q18	7.77	0.735
Q19	7.74	0.729
Q20	7.76	0.733
Q21	7.74	0.730
Q22	7.78	0.733
Q23	7.74	0.731
Q24	7.68	0.727
Q25	7.73	0.730
Q26	7.61	0.728
Q27	7.5	0.732
